# PDGF-BB and IL-4 co-overexpression is a potential strategy to enhance mesenchymal stem cell-based bone regeneration

**DOI:** 10.1186/s13287-020-02086-8

**Published:** 2021-01-07

**Authors:** Ning Zhang, Chi-Wen Lo, Takeshi Utsunomiya, Masahiro Maruyama, Ejun Huang, Claire Rhee, Qi Gao, Zhenyu Yao, Stuart B. Goodman

**Affiliations:** 1 Department of Orthopaedic Surgery, Stanford University, Stanford, CA USA; 2 Department of Bioengineering, Stanford University, Stanford, CA USA

**Keywords:** Interleukin-4, Platelet-derived growth factor, Co-overexpression, Genetically modified mesenchymal stem cells, Bone regeneration

## Abstract

**Background:**

Mesenchymal stem cell (MSC)-based therapy has the potential for immunomodulation and enhancement of tissue regeneration. Genetically modified MSCs that over-express specific cytokines, growth factors, or chemokines have shown great promise in pre-clinical studies. In this regard, the anti-inflammatory cytokine interleukin (IL)-4 converts pro-inflammatory M1 macrophages into an anti-inflammatory M2 phenotype; M2 macrophages mitigate chronic inflammation and enhance osteogenesis by MSC lineage cells. However, exposure to IL-4 prematurely inhibits osteogenesis of MSCs in vitro; furthermore, IL-4 overexpressing MSCs inhibit osteogenesis in vivo during the acute inflammatory period. Platelet-derived growth factor (PDGF)-BB has been shown to enhance osteogenesis of MSCs with a dose-dependent effect.

**Methods:**

In this study, we generated a lentiviral vector that produces PDGF-BB under a weak promoter (phosphoglycerate kinase, PGK) and lentiviral vector producing IL-4 under a strong promoter (cytomegalovirus, CMV). We infected MSCs with PDGF-BB and IL-4-producing lentiviral vectors separately or in combination to investigate cell proliferation and viability, protein expression, and the capability for osteogenesis.

**Results:**

PDGF-BB and IL-4 co-overexpression was observed in the co-infected MSCs and shown to enhance cell proliferation and viability, and osteogenesis compared to IL-4 overexpressing MSCs alone.

**Conclusions:**

Overexpression of PDGF-BB together with IL-4 mitigates the inhibitory effect of IL-4 on osteogenesis by IL-4 overexpressing MSCS. PDGF-BB and IL-4 overexpressing MSCs may be a potential strategy to facilitate osteogenesis in scenarios of both acute and chronic inflammation.

## Background

MSC-based therapy is a promising strategy for immunomodulation and enhancement of tissue regeneration. In fact, MSCs have been identified as a reserve for skeletal tissue regeneration [1]. However, specific properties of MSCs, such as the capability for differentiation and potential for immunomodulation, need further refinement to optimize MSC-based therapy [2]. In this regard, genetically modified MSCs have become a potential strategy for MSC-based therapy. One such approach is over-expression of an immunomodulatory and anti-inflammatory cytokine, such as interleukin-4 (IL-4). IL-4 converts pro-inflammatory M1 macrophages into an anti-inflammatory, tissue regenerative M2 phenotype. Modulation of M1 to M2 phenotype at an appropriate time optimized the osteogenic differentiation of MSCs in vitro [3]. However, IL-4 inhibits MSC-mediated bone formation [3–5] during the acute (as opposed to the chronic) inflammatory period of bone healing, which normally lasts several days. A novel strategy of genetically modified MSC therapy to optimize bone regeneration and remodeling is continuous production of IL-4 by genetically modified MSCs together with a second growth factor to counteract the initial potential adverse effects of IL-4 during the first few days of acute inflammation.

Platelet-derived growth factor (PDGF)-BB stimulates MSCs, resulting in enhanced angiogenesis and osteogenesis with a dose-dependent effect [6, 7]. Osteogenesis was enhanced by relatively low doses of PDGF-BB in vitro; in vivo studies showed that trabecular bone formation and trabecular connectivity were increased by overexpression by the lentiviral vector of PFGF-BB under a relatively week PGK promoter, completely avoiding osteomalacia and secondary hyperparathyroidism seen with higher doses [8].

We have generated IL-4 secreting MSCs using a lentiviral vector under a relatively strong CMV promotor [5, 9]. In the current study, we constructed a lentiviral vector that produced PDGF-BB under a weak (phosphoglycerate kinase; PGK) promoter [8]. MSCs were infected with the PDGF-BB-producing lentiviruses in combination with the IL-4 lentiviral vector [5] to generate IL-4 only overexpressing MSCs, PDGF-BB only overexpressing MSCs, and co-overexpressing IL-4 and PDGF-BB MSCs populations. We also added recombinant PDGF-BB protein to IL-4 overexpressing MSC cultures as another method of treatment, to more clearly establish the effects of PDGF protein. We investigated cell proliferation and cell viability and the capacity for osteogenesis of these different MSCs constructs to test whether the co-overexpression of IL-4 and PDGF-BB was superior to single gene over-expression MSCs for the purposes of enhancement of osteogenesis.

## Material and methods

### Cells

Isolation and characterization of the male BALB/c and C57BL/6 murine bone marrow-derived MSCs have been described previously [10]. Stanford’s Administrative Panel on Laboratory Animal Care (APLAC) approved this isolation protocol and Institutional Guidelines for the Care and Use of Laboratory Animals were observed in all aspects of this project. Briefly, bone marrow was collected from femurs and tibias of 8- to 10- week-old BALB/c and C57BL/6 male mice. The bone marrow with cells was carefully suspended and filtered through 70-μm cell strainer, spun down and resuspended in α-minimal essential medium (α-MEM, Thermo Fisher Scientific, Waltham, MA USA) supplied with 10% certified fetal bovine serum (FBS, Invitrogen, Thermo Fisher Scientific, Waltham, MA USA) and antibiotic-antimycotic solution (100 units of penicillin, 100 μg of streptomycin, and 0.25 μg of amphotericin B per milliliter, Hyclone, Thermo Fisher Scientific, Waltham, MA USA). Medium was replaced the next day to remove unattached cells (passage 1). Immunophenotype of isolated MSCs: spinocerebellar ataxia type 1 (Sca1+)/CD105+/CD44+/CD45−/CD34−/CD11b− was characterized by flow cytometry (LSRII, Stanford Shared FACS Facility, Stanford, CA, USA) at passage 4. Identified MSC passages 4–8 were used to conduct the experiments.

### Construction of plasmids

The constitutive mouse IL-4 expression lentivirus driven by cytomegalovirus (CMV) promoter was released from the IL-4 expression plasmid pCMV3-mIL4 (Sino Biological Inc.) by digestion with SpeI/NotI restriction enzyme and ligated into the pCDH-CMV-copGFP lentiviral expression vector (CD511B-1; System BiosciencesPalo Alto, CA) to generate the pCDH-CMV-IL-4-copGFP vector [5]. The fragment containing the human PDGF-BB element with EcoRI and BamHI cutting sites was amplified by polymerase chain reaction (PCR; Forward primer: 5′-ggagaattcatgaatcgctgctgggcg-3′; Reverse primer: 5′-ccgggatccctaggctccaagggtctc-3′) from the plasmid pBabe-PDGF/B-zeo (Plasmid #17757, Addgene, Watertown, MA, USA) using Phusion high-fidelity DNA polymerase (NEB, Ipswich, MA, USA) then digested by EcoRI/BamHI and ligated into the pCDH-CMV-MCS-mRFP lentiviral expression vector to generate the pCDH-CMV-PDGF-BB-mRFP vector. The fragment containing weak phosphoglycerate kinase (PGK) promoter with cutting sites was obtained from pCDH-PGK (Plasmid #72268, Addgene, Watertown, MA, USA) by digestion with EcoRI/HpaI restriction enzyme and replaced the CMV promoter in pCDH-CMV-PDGF-mRFP lentiviral expression vector by with EcoRI/HpaI digestion and ligation to generated pCDH-PGK-PDGF-mRFP lentiviral expression vector. Competent *Escherichia coli* bacterial cells (One Shot™ Stbl3™ Chemically Competent *E. coli*; Invitrogen, Thermo Fisher Scientific, Waltham, MA USA) were used to obtain the final vector after the ligation.

### Generation of genetically modified MSCs

**Fig. 1:**
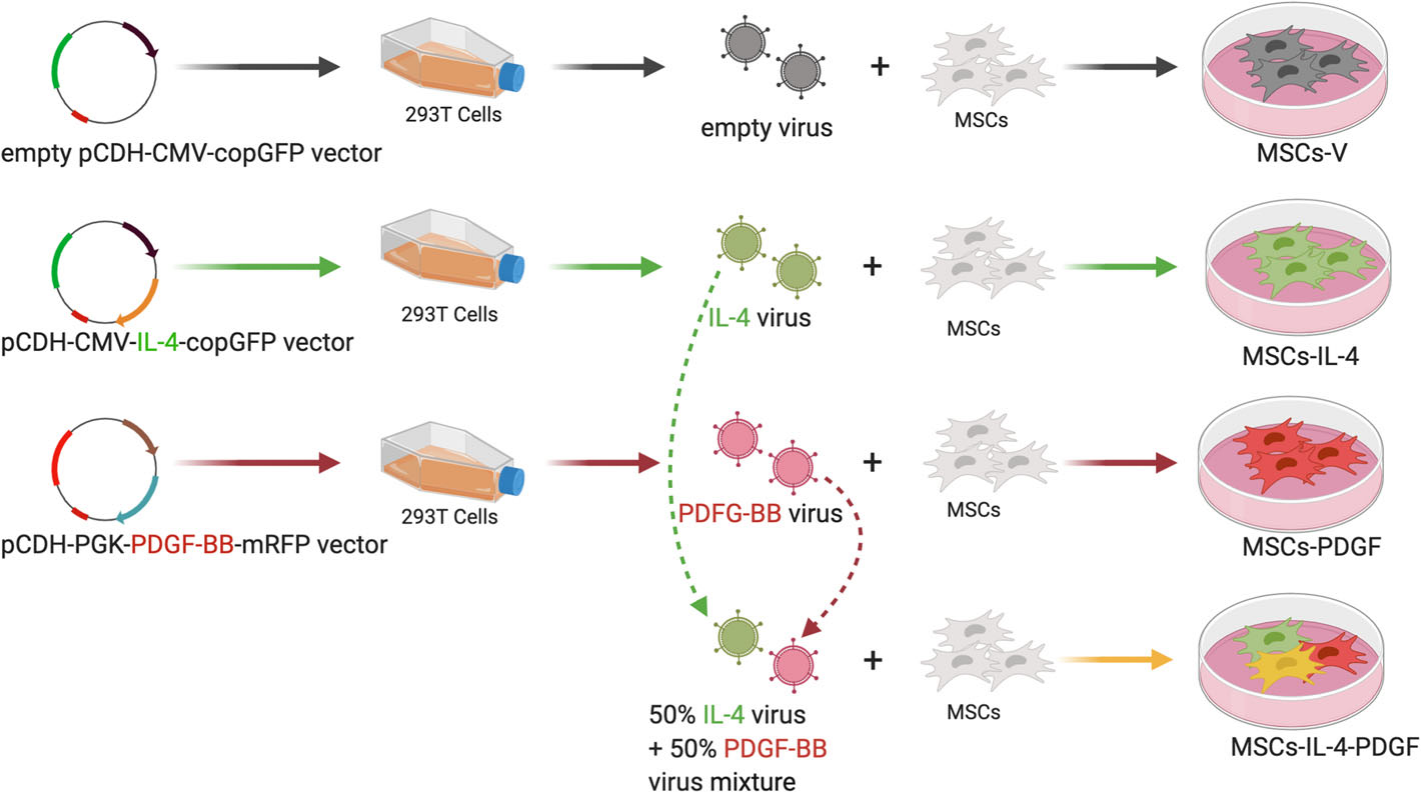
Experimental design to generate the genetically modified MSCs

The lentiviral vector preparation was performed as previously described [11]. Human embryonic kidney 293T cells (ATCC, Manassas, VA) were used to co-transfect the control lentivirus vector pCDH-CMV-copGFP or the mouse IL-4 secreting pCDH-CMV-mIL-4-copGFP expressing lentivirus vector [5] or the human PDGF-BB secreting pCDH-PGK-hPDGF-BB-mRFP expressing lentivirus vector together with psPAX2 packaging vector and pMD2G VSV-G envelope vector using the calcium phosphate transfection kit (Clontech, Mountain View, CA) with 25 μM chloroquine. The virus was diluted in MSCs’ culture medium supplemented with 6 μg/mL of polybrene (Sigma Aldrich, St. Louis, MO, USA) and infected to murine MSCs at multiplicity of infection (MOI) = 100 (Fig. 1, Table 1). Eight groups were included in this study (Table 1): (1) MSCs, unaltered MSCs; (2) MSCs-V, MSCs infected with empty lentivirus vector; (3) MSCs-IL-4, MSCs infected with mIL-4 secreting lentivirus vector; (4) MSCs-PDGF, MSCs infected with PDGF-BB secreting lentivirus vector; (5) MSCs-IL-4-PDGF, MSCs co-infected with both mIL-4 and PDGF-BB secreting lentivirus vectors with half dosage in single infection respectively; (6) MSCs+(PDGF), unaltered MSCs without genetically modification cultured with 0.5 ng/ml recombinant hPDGF-BB protein in the medium; (7) MSCs-V+(PDGF), MSCs infected with empty lentivirus vector cultured with 0.5 ng/ml recombinant hPDGF-BB protein in the medium; and (8) MSCs-IL-4+(PDGF), MSCs infected with mIL-4 secreting lentivirus vector cultured with 0.5 ng/ml recombinant hPDGF-BB protein in the medium. The infected cells were GPF positive confirmed by fluorescence microscope (Keyence, Itasca, IL, USA) and flow cytometry (LSRII, Stanford Shared FACS Facility, Stanford, CA, USA) 3 days post-infection.

**Table 1:** Different treatments and experimental design

Group	Genetically modified method	Treatment
MSCs	N/A	Culture medium/osteogenic medium
MSCs-V	Infected with empty control lentivirus vector	Culture medium/osteogenic medium
MSCs-IL-4	Infected with mIL-4 secreting lentivirus vector	Culture medium/osteogenic medium
MSCs-PDGF	Infected with hPDGF-BB secreting lentivirus vector	Culture medium/osteogenic medium
MSCs-IL4-PDGF	Co-infected with both mIL-4 and hPDGF-BB lentivirus vectors, half of the dosage used in single infection respectively	Culture medium/osteogenic medium
MSCs+(PDGF)	N/A	Culture medium/osteogenic medium + 0.5 ng/ml recombinant hPDGF-BB protein
MSCs-V+(PDGF)	Infected with control lentivirus vector	Culture medium/osteogenic medium + 0.5 ng/ml recombinant hPDGF-BB protein
MSCs-IL-4+(PDGF)	Infected with mIL-4 secreting lentivirus vector	Culture medium/osteogenic medium + 0.5 ng/ml recombinant hPDGF-BB protein

### Cell proliferation assay

Six hundred forty-seven EdU Click Proliferation Kit (BD, San Jose, CA, USA) was used to assay the proliferation carefully following the manufacturer’s protocol. Briefly, cells were seeded in 96-well plates at 1000 cells per well in culture medium with 10 μM/ml EdU (*n* = 3 for each group). Cells were cultured for 7 days then washed with BD Pharmingen™ Stain Buffer (BSA) (BD, San Jose, CA, USA) and fixed with Fixative Solution (4% paraformaldehyde-based, component of 647 EdU Click Proliferation Kit); EdU in the cells was detected using a fluorescence microscope (Keyence, Itasca, IL, USA). EdU-positive Cell numbers were counted and calculated in same-size and random selected areas near the center of the original images of 3 experimental replicate wells using ImageJ (“analyze particles” function).

### Cell viability assay

Viability of the MSCs was tested by CellTiter-Blue Cell Viability Assay (Promega, Madison, WI, USA) according to manufacturers’ protocol. Briefly, MSCs were seeded in 96-well plates at 1000 cells per well in 100 μl culture medium and cultured for 1, 3, 5, and 7 days (*n* = 6 for each group of each time point). Viability of MSCs was tested at each time point by adding 20 μl CellTiter-Blue® Cell Viability assay reagent. The plate was shaken for 10 s and the cells were incubated under standard cell culture conditions for 1 h; then, the plate was shaken for 10 s and fluorescence recorded at 560_Ex_/590_Em_ by SpectraMax M2e Microplate Readers (Molecular Devices, San Jose, CA, USA).

### Enzyme-linked immunosorbent assay (ELISA)

ELISA kits for mouse IL-4 and human PDGF-BB (R&D system, Minneapolis, MN, USA) were used to quantitate IL-4 and PDGF-BB expression by the genetically modified MSCs. The manufacturers’ protocols were carefully followed. The optical densities were determined using SpectraMax M2e Microplate Readers (Molecular Devices, San Jose, CA, USA) set at 450 nm with wavelength correction set to 540 nm.

### Osteogenesis assay

MSCs were seeded at a density of 1 × 10^4^ per well in a 24-well plate and were cultured in osteogenic medium (α-MEM supplemented with 10% FBS, 100 nmol/L dexamethasone, 10 mmol/L β-glycerol phosphate, and 50 μmol/L ascorbate-2-phosphate). Alkaline phosphatase (ALP) activity at week 2 was detected by 1-Step™ NBT/BCIP Substrate Solution (Thermo Fisher Scientific, Waltham, MA USA) staining and the positive area ratio of the whole well was analyzed using ImageJ (Color threshold: Hue 150-240; Saturation 15-255; Brightness 0-255). Extracellular matrix mineralization in mouse MSCs was stained using alizarin red S (Sigma Aldrich, St. Louis, MO, USA) at week 4. The results were photographed, and the staining was eluted by 10% cetylpyridinium chloride (Sigma Aldrich, St. Louis, MO, USA) and measured absorbance at 562 nm.

### Statistical analysis

All data presented in this study were expressed in mean ± standard deviation (SD). One-way ANOVA with Tukey’s post hoc testing was conducted using Prism 8 (GraphPad Software, San Diego, CA, USA). A *p* value of < 0.05 was regarded as statistically significant.

## Results

### PDGF-BB and IL-4 co-overexpression is successfully generated in co-infected MSC group

**Fig. 2:**
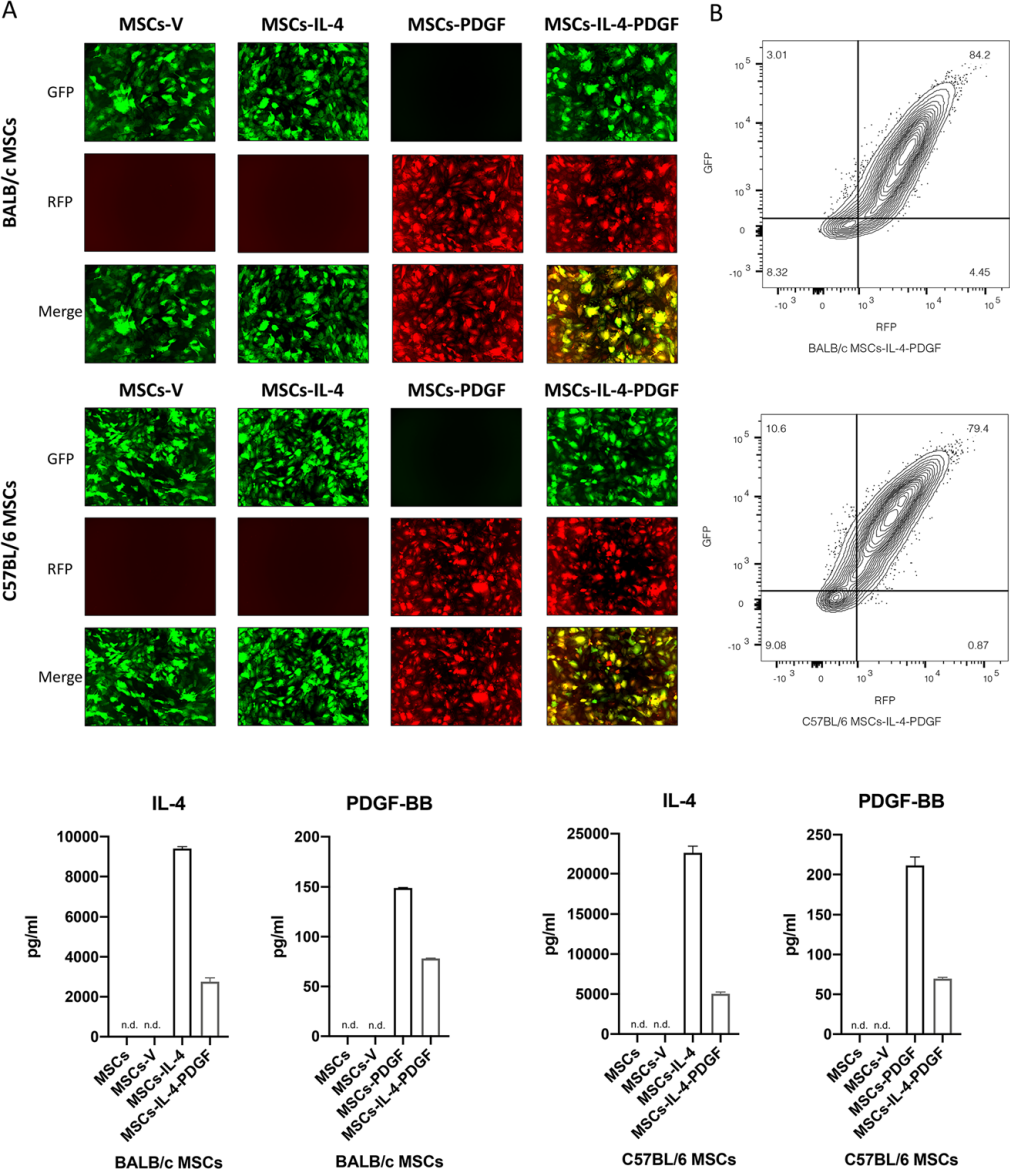
Characterization of infected MSCs by IL-4 and/or PDGF-BB lentiviral vectors and the expression level of IL-4 and PDGF-BB of these MSCs. **a** Representative fluorescence microscopy images of MSCs infected by lentiviral vectors. GM MSCs are GFP/RFP positive. **b** Characterization of MSCs-IL-4-PDGF groups, GFP, and RFP was analyzed by flow cytometry. **c** IL-4 and PDGF-BB secreting level detected by ELISA in the GM MSCs culture medium for 1-day culture. (n.d.: Cannot be detected by ELISA). “MSCs”, MSCs without infection; “MSCs-V”, MSCs infected with empty control lentivirus vector; “MSCs-IL-4”, MSCs infected with mIL-4 secreting lentivirus vector; “MSCs-PDGF”, MSCs infected with hPDGF-BB secreting lentivirus vector; “MSCs-IL-4-PDGF”, MSCs co-infected with both mIL-4 and hPDGF-BB lentivirus vectors, half of the dosage used in single infection respectively

MSCs were successfully infected by empty vector or single and combined use of lentiviral vectors expressing IL-4 and PDGF-BB. Infected cells showed fluorescence (GFP/RFP) positivity (Fig. 2a, b). 84.2% of BALB/c MSCs and 79.4% of C57BL/6 MSCs in MSCs-IL-4-PDGF group were co-infected by both IL-4 and PDGF-BB lentiviral vectors (Fig. 2b). IL-4 and PDGF-BB secretion levels in the culture medium supernatant were assayed by ELISA. IL-4 and PDGF-BB secretion in the unaltered MSCs group and empty control vector infected MSCs (MSCs-V) group were below the detectable range of ELISA. In both BALB/c and C57BL/6 MSCs, IL-4 expression (Fig. 2c) was higher in MSCs infected with IL-4 expressing lentiviral vector (MSCs-IL-4) compared to MSCs co-infected with IL-4 and PDGF-BB lentiviral vectors (MSCs-IL-4-PDGF); however, based on a previous study, the IL-4 expression level was in the therapeutically effective range in both groups (from 172.18–3679.95 pg/ml for MSCs infected using a relatively weak promoter lentiviral vector and 19,415.5–22,291.0 pg/ml for MSCs infected using strong promoter lentiviral vector) [5]. A slight reduction of PDGF-BB expression was also found in the MSCs-IL-4-PDGF group compared to the MSCs-PDGF group in both BALB/c and C57BL/6 MSCs (Fig. 2c), but levels in both strains were also in the therapeutically effective range of expression (adding 0.3 and 3 ng/ml recombination PDGF-BB showed enhanced bone nodule formation, 30 ng/ml recombination PDGF-BB showed reduced bone nodule formation, gene modified MSCs using a relatively weak promotor PGK as we used in this study showed increased bone strength) [8].

### Cell proliferation and cell viability were enhanced in the presence of PDGF-BB

**Fig. 3:**
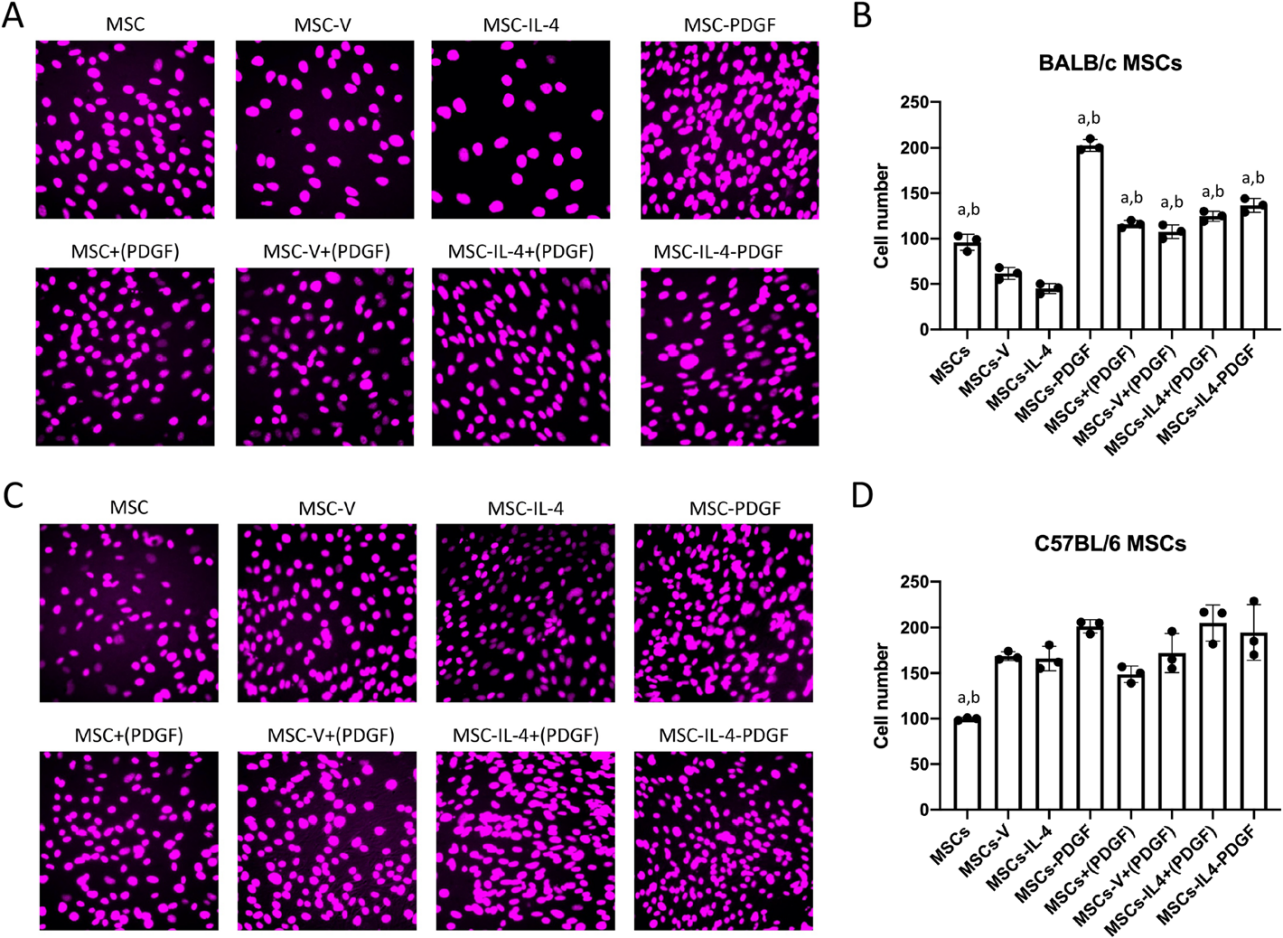
Cell proliferation showed by EdU staining and quantitative assay in all groups in day 7 after infection. Representative fluorescence microscopy images (**A**) and cell number (**B**) of BALB/c MSCs stained by EdU. Representative fluorescence microscopy images (**C**) and cell number (**D**) of C57BL/6 MSCs stained by EdU. Same fixed size of area was selected randomly near the center of the original images of 3 experimental replicate wells. Cell number was count automatically with manually correction using ImageJ (“analyze particles” function). a, *p* value < 0.05 compared to MSCs-V group; b, *p* value < 0.05 compared to MSCs-IL-4 group. “+(PDGF)”, add 0.5 ng/ml hPDGF-BB recombinant protein in the culture medium while culturing the cells

Cell proliferation of the MSCs in both BALB/c and C57BL/6 MSCs was examined (Fig. 3), to determine the effects of PDGF-BB on cell proliferation. In BALB/c MSCs, cell proliferation was reduced after infection by IL-4 (MSCs-IL4) and the control (MSCs-V) vectors, compared to MSCs; however, cell proliferation was significantly enhanced in MSCs-PDGF and MSCs-IL-4-PDGF groups compared to MSC-IL4 group (Fig. 3A, B). Significant enhancement was also observed in the groups in which human PDGF-BB recombinant protein was added [MSCs+(PDGF), MSCs-V+(PDGF) and MSCs-IL-4+(PDGF) groups] compared to MSCs-IL4 and MSCs-V groups. Similar trends were observed with C57BL/6 MSCs even though no significant differences were detected. Interestingly, the cell proliferation of MSCs was lower compared to MSCs-V and MSCs-IL-4 groups (Fig. 3C, D).

**Fig. 4:**
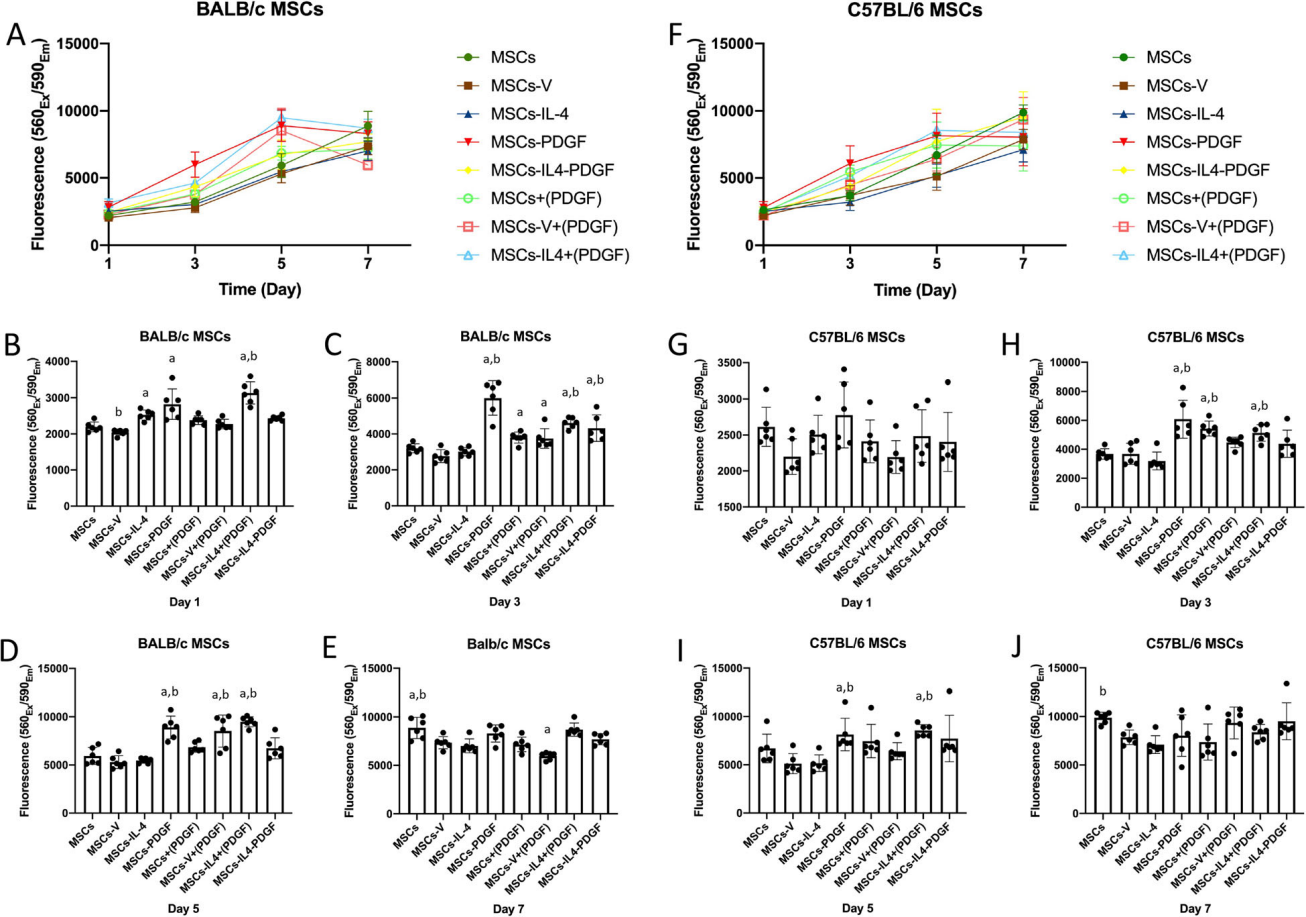
Cell viability at days 1, 3, 5, and 7 after infection. Cell viability in all groups (**A**) at all time points and at days 1 (**B**), 3 (**C**), 5 (**D**), and 7 (**E**) of BALB/c MSCs; cell viability in all groups (**F**) at all time points and at days 1 (**G**), 3 (**H**), 5 (**I**), and 7 (**J**) of C57BL/6 MSCs. a, *p* value < 0.05 compared to MSCs-V group, b, *p* value < 0.05 compared to MSCs-IL-4 group

We also studied the influence of PDGF-BB on cell viability in BALB/c and C57BL/6 MSCs. In BALB/c MSCs, the addition of PDGF-BB enhanced cell viability compared with the MSCs-IL-4 group (Fig. 4A). At day 1, cell viability of the MSCs-IL-4 group and MSCs-PDGF was significantly higher than the MSCs-V group; a trend for enhancement was also observed in MSCs-PDGF group compared with MSCs-IL-4 group. Adding recombinant PDGF-BB protein to the MSCs-IL-4+(PDGF) group dramatically increased cell viability compared with MSCs-IL-4 group (Fig. 4B). At day 3, a significant increase was detected in MSCs-PDGF, MSC-IL-4-PDGF compared with MSCs-IL-4 and MSCs-V groups. Adding recombinant PDGF-BB protein MSCs+(PDGF) and MSCs-V+(PDGF) groups enhanced cell viability compared with the MSCs-V group. Cell viability of MSCs- IL-4+(PDGF) group was increased dramatically compared with the MSCs-IL-4 group (Fig. 4C). At day 5, cell viability of MSCs-PDGF was significantly higher than MSCs-V and MSCs-IL-4 groups. The same significant enhancement was also detected in MSCs-V+(PDGF) and MSCs-IL-4 + (PDGF) groups. Cell viability of MSC-IL-4-PDGF was also higher than the MSCs-IL-4 group but was not statistically significant. At day 7, MSCs had significantly higher cell viability than MSCs-V and MSCs-IL-4. Generally, relative changes among groups became smaller over time. In C57BL/6 MSCs, a similar situation was observed from day 1 to day 7. At day 1, a similar trend was detected without significant difference. At day 3, cell viability of MSCs-PDGF, MSCs+(PDGF), and MSCs-IL-4+(PDGF) was significantly higher than that of the MSCs-V and MSCs-IL-4 groups. Cell viability of the MSCs-IL-4-PDGF group was also higher than that of the MSCs-IL-4 group. At day 5, cell viability of MSCs-PDGF was significantly higher than MSCs-V and MSCs-IL-4 groups. Similar enhancement was also detected in MSCs-IL-4+(PDGF) groups. MSC-IL-4-PDGF also showed higher cell viability than the MSCs-IL-4 group but did not reach statistical significance. At day 7, MSCs-IL-4 had significantly lower cell viability than MSCs.

### Osteogenesis was enhanced in the presence of PDGF-BB

**Fig. 5:**
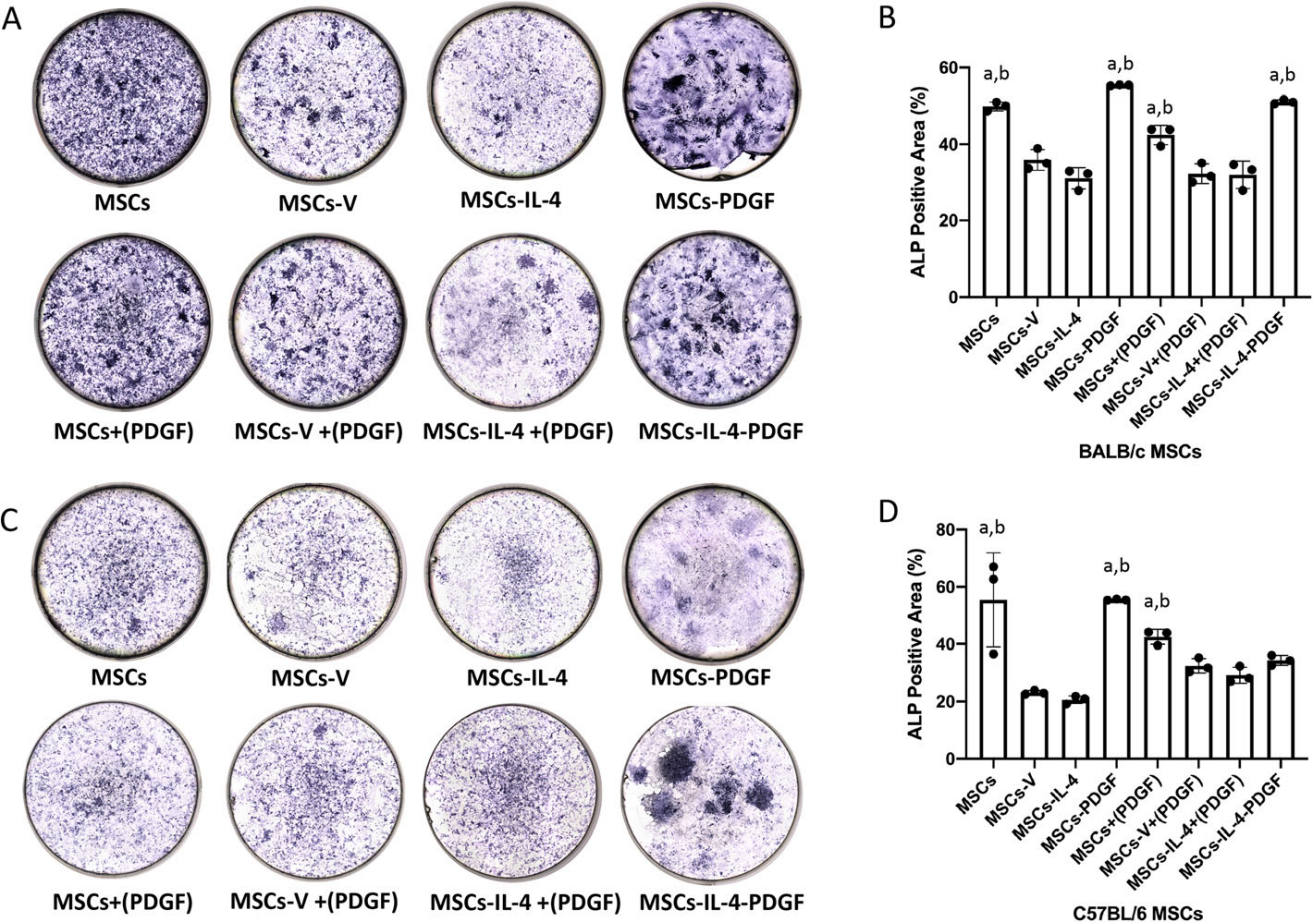
ALP staining in all groups after 14 days of osteogenic differentiation. Representative images of ALP staining (**A**) in all groups and quantitative of the ALP positive area (**B**) in all groups of BALB/c MSCs. Representative images of ALP staining (**C**) in all groups and ALP level (**D**) in all groups of C57BL/6 MSCs

Osteogenesis for all groups was examined using osteogenic medium in the cell cultures. In BALB/c MSCs, the ALP staining was decreased in the MSCs-IL-4 and MSCs-V groups compared to the MSCs group; ALP staining was enhanced in the MSCs-PDGF and MSCs-IL-4-PDGF groups compared to the MSCs-IL4 group (Fig. 5A). Quantitative assessment of the ALP-positive area confirmed these findings (Fig. 5B). MSCs-V+(PDGF) group was also enhanced compared with the MSCs-V group. In C57BL/6 MSCs, the same significant enhancement of MSCs-PDGF and MSCs+(PDGF) was detected. Although no significant difference between MSCs-IL-4-PDGF and MSCs-IL-4 was detected, clear enhancement of the ALP staining and positive area in the quantitative assay can be observed (Fig. 5A, B).

**Fig. 6:**
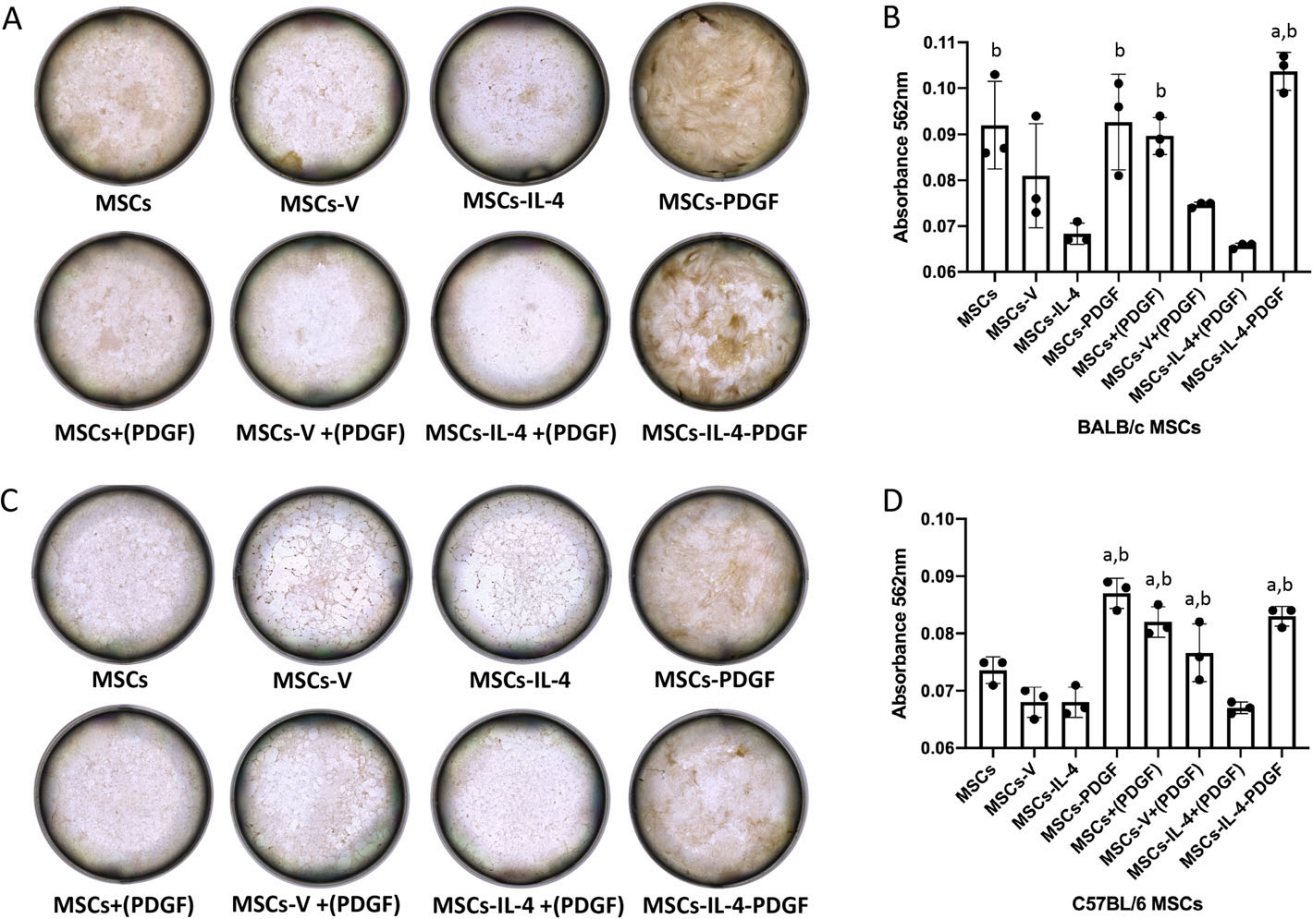
Alizarin Red S staining in all groups after 28 days of osteogenic differentiation. Representative images of Alizarin Red S staining (**A**) in all groups and quantified by absorbance at 562 nm (**B**) after elution in all groups of BALB/c MSCs. Representative images of Alizarin Red S staining (**C**) in all groups and quantified by absorbance at 562 nm (**D**) after elution in all groups of C57BL/6 MSCs

The Alizarin Red S staining further confirmed our findings concerning the enhancement of osteogenic differentiation in the presence of PDGF-BB. In BALB/c MSCs, the mineralization was significantly decreased in the MSCs-IL-4 group compared to the MSCs group; and significant enhancements were observed in the MSCs-PDGF and MSCs-IL-4-PDGF groups compared to MSCs-IL4 (Fig. 6A). Quantitative assessment of the Alizarin Red S staining confirmed these findings (Fig. 6B). In C57BL/6 MSCs, the mineralization was also decreased in the MSCs-IL-4 group compared to the MSCs group without significant difference. Significant enhancement of MSCs-PDGF, MSCs-IL-4-PDGF, MSCs-V+(PDGF), and MSCs+(PDGF) groups compared to either MSCs-IL4 or MSCs-V groups were found (Fig. 6A, B). The percentage of fluorescence-positive cells decreased less than 10% after 2 weeks of culture and ~ 20% after 4 weeks of culture, which might be due to the osteogenic differentiation process. As the integration of the target gene by lentivirus was stable and theoretically the proliferation will be stopped after culturing in osteogenic medium, the percentage of transduced cells that survived at 2 or 4 weeks would be about the same.

## Discussion

This study showed that co-overexpression of PDGF-BB and IL-4 in murine MSCs enhanced cell proliferation, cell viability, and osteogenesis compared to IL-4 overexpressing MSCs alone. These findings clearly demonstrate that PDGG-BB co-expression together with IL-4 was able to overcome the initial inhibitory effect of IL-4 overexpressing MSCs on osteogenesis. Thus, these results highlight a potential strategy to facilitate osteogenesis using genetically modified PDGF-BB and IL-4 overexpressing MSCs in the scenarios of both acute and chronic inflammation.

Controlled or continuous release of cytokines, growth factors, or chemokines that promote osteogenesis at the region of interest is critical for bone regeneration. Although biomaterials have been used as carriers for these soluble factors, local delivery remains a problem due to the biodegradability of the materials, release profiles, and potential for adverse inflammatory and immunological reactions [12, 13], often leading to a short biological half-life. Recently, novel scaffold technologies for delivery of cells and biologics which could protect the cells as well as stimulate endogenous recruitment of cells to support osteogenesis via controlled delivery of biologics have been developed [12, 14–16]. However, as an alternative strategy, regional gene therapy could not only provide a time- and dose-controlled delivery of growth factors, cytokines, or chemokines for inducing bone formation [9, 17, 18] but also provide the source of potential cells that could facilitate the process more efficiently. The strategy of genetically modified cells appears to be a viable and more sustained biomaterial drug delivery system for bone regeneration [12, 15, 16].

MSCs have been applied as gene carriers to enhance the therapeutic capability in tissue regeneration and treatments of various diseases [19]. Genetically modified MSCs that overexpress IL-4 modulated inflammatory macrophages into an anti-inflammatory phenotype [20] which augmented osteogenesis of MSCs in coculture with macrophages [3, 21, 22]. Inflammation is the first stage of bone formation and fracture healing. Continuous IL-4 overexpression by genetically modified MSCs could impair bone formation during the first few days after injury, during the acute inflammatory phase [4, 21]; in fact, in vitro and in vivo studies have demonstrated this adverse effect [5, 23]. The purpose of this study was to generate genetically modified MSCs that overexpress IL-4 in combination with PDGF-BB; this construct could counteract the potentially adverse effects of IL-4 on bone healing in the acute inflammatory phase, mitigate chronic inflammation, and therefore enhance the proliferation of MSCs and osteogenesis in both scenarios. This strategy for the treatment of inflammatory diseases of bone is a novel approach for bone tissue regeneration in scenarios of both acute and chronic inflammation.

Previous studies on gene therapy have focused on a single specific cytokine, growth factor, or chemokine. However, bone regeneration requires interactions among multiple cell types via a plethora of cytokines and other substances in a specific temporal sequence. Fang et al. [24] showed accelerated bone formation by using the combined expression of two interacting genes (BMP-4 and hPTH 1-34) compared to either factor alone. Others also have shown that combinations of regenerative molecules by transduction of two or three osteoprogenitor cell lines expressing BMP-2, BMP-4 or BMP-7 resulted in greater bone formation compared to using transduction methods with a single factor [25, 26]. These studies support the potential use of cells with combinations of co-expressing cytokines, growth factors, or chemokines to enhance the bone formation.

The described co-infection method with IL-4 and PDGF-BB lentiviral vectors was used to generate the genetically modified MSCs populations. These two lentiviral vectors were mixed using half the titer of single infection and infected the MSCs simultaneously which generated the genetically modified MSC populations within a shorter time and improved cell survival. Previous study of C57BL/6 MSCs infection with single lentivirus using different MOI showed that using half of the titer also reached 70% efficiency without affecting the viability [27]. The co-infected MSCs showed a dominant proportion in the genetically modified MSCs co-infected by IL-4 and PDGF-BB lentiviral vectors. These cell populations also secreted significant amounts of IL-4 and PDGF-BB proteins of which both were in the therapeutically effective range [5, 8]. The secretion of IL-4 by the co-infected MSC populations was slightly decreased compared with the single infected MSCs. Leakage of IL-4 into the systemic circulation could potentially increase the risk of infection [28, 29], allergic reactions [30], and synovial hyperplasia [31]. Previous research using novel NF-kB sensing IL-4 secreting MSCs which had similar expression levels with the genetically modified co-infected MSCs in this study could potentially mitigate inflammation-associated settings and reduce the adverse effects of excessive IL-4 secretion [5, 20]. Constitutive promoters of lentiviral vectors were widely used in genetically modified MSCs to obtain sufficient doses of the target proteins [32]; Strong CMV promotor of IL-4 vector [5, 9] and relatively weak PGK promotor in PDGF-BB vector [8] were used in this study to generate genetically modified MSCs based on previous studies. By using continuously overexpressing genetically modified MSCs, we have generated more direct in vitro evidence concerning the capabilities of genetically modified MSCs co-expressing IL-4 and PDGF-BB.

We used two murine strains of MSCs in the current study to investigate the co-expression of genetically modified MSCs as MSCs isolated from different strains of inbred mice vary in their rates of proliferation and differentiation potential [33]. It is been reported that PDGF-BB enhanced the proliferation of several cell types including endothelial cells [6] and populations of MSCs [34, 35]. By studying the cell proliferation and cell viability, the results showed that the presence of PDGF-BB from genetically modified MSCs or exogenous PDGF-BB protein in BALB/c MSCs increased cell proliferation and cell viability. Similar trends were observed in C57BL/6 MSCs but the changes were less dramatic. It is very interesting that cell proliferation of BALB/c MSCs was decreased even after infection with the empty control lentiviral vector and IL-4 secreting lentiviral vector, but C57BL/6 MSCs showed increased proliferation after single infection with these two vectors. These differences might be due to the differences in the murine strains of the MSCs. Despite the above, the enhancement of cell proliferation and viability by PDGF-BB was observed in both cell strains.

Recombinant human PDGF-BB (rhPDGF-BB) in combination with an osteoinductive tricalcium phosphate (TCP)-collagen matrix has been applied clinically in ankle and hindfoot arthrodesis and was shown to be a safe and effective alternative to autograft [36, 37]. In vitro and in vivo studies have previously demonstrated the capacity for PDGF-BB to facilitate osteogenesis by stem cells [7, 34, 35]. Furthermore, PDGF-BB secreted by preosteoclasts induced angiogenesis during coupling with osteogenesis [6]. Human periodontal stem cells overexpressing PDGF-BB showed enhanced osteogenesis and aided the repair of alveolar bone defects [38]. Chen et al. [8] found a dose-dependent effect of PDGF-BB on osteogenic capacity of MSCs; low dosage (0.3 ng/ml) of PDGF-BB could enhance osteogenesis of MSCs in vitro and overexpressing PDGF-BB under a weak promotor could enhance bone formation in vivo *without* causing osteomalacia and secondary hyperparathyroidism seen with higher doses.

The presence of PDGF-BB overexpressing genetically modified MSCs enhanced both ALP activity and matrix mineralization. In our study, the addition of recombinant PDGF-BB also rescued the osteogenesis by the MSCs and MSCs-V groups but did not enhance osteogenesis in the MSCs-IL-4 groups (Figs. 5 and 6). Based on this observation, overexpressing PDGF-BB in genetically modified MSCs was a better option compared to adding recombinant PDGF-BB protein to the culture medium for osteogenesis.

This study also has some limitations. In the current study, we generated the PDGF-BB and IL-4 co-overexpression genetically modified MSC populations using lentiviral vectors with fluorescence markers simply and confirmed the expression of PDGF-BB and IL-4 from this MSCs populations. Although the co-infected MSC population is the dominant part in the group, the comparisons of simultaneous and sequential infection with different proportions might generate a clearer population of genetically modified MSCs. Continuous overexpression IL-4 and PDGF-BB genetically modified MSCs has provided direct in vitro evidence concerning the genetically modified MSCs’ co-expression of IL-4 and PDGF-BB. In order to monitor potential adverse clinical effects of excessive IL-4 and PDGF-BB, an in vivo study would be prudent. Further investigation will also be conducted using inflammation-regulated genetically modified MSCs such as with NF-kB sensing [5, 20]. In vivo confirmation of these findings of using genetically modified MSCs in animal models of inflammatory bone loss [39] and nonunion in critical-size bone defects [40] are needed to confirm these in vitro observations.

## Conclusions

Genetically modified MSCs that overexpress PDGF-BB with IL-4 enhanced cell proliferation, viability, and osteogenesis, compared to IL-4 overexpressing MSCs alone. Thus, MSCs that overexpress both PDGF-BB and IL-4 may be a potential strategy to facilitate osteogenesis in scenarios of both acute and chronic inflammation.

## Data Availability

All data generated or analyzed during this study are included in this published article.

## Abbreviations

MSCs: Mesenchymal stem cells
PDGF-BB: Platelet-derived growth factor-BB
IL-4: Interleukin-4
PGK: Phosphoglycerate kinase
CMV: Cytomegalovirus
MOI: Multiplicity of infection
ALP: Alkaline phosphatase
GFP: Green fluorescent protein
RFP: Red fluorescence protein
BMP: Bone morphogenetic protein
hPTH: Human parathyroid hormone
TCP: Tricalcium phosphate
